# Astrocytes of the Anterior Commissure Regulate the Axon Guidance Pathways of Newly Generated Neocortical Neurons in the Opossum *Monodelphis domestica*

**DOI:** 10.3390/ijms25031476

**Published:** 2024-01-25

**Authors:** Katarzyna Bartkowska, Paulina Koguc-Sobolewska, Ruzanna Djavadian, Krzysztof Turlejski

**Affiliations:** 1 Nencki Institute of Experimental Biology, Polish Academy of Sciences, 02-093 Warsaw, Poland; k.bartkowska@nencki.edu.pl (K.B.); p.koguc-sobolewska@nencki.edu.pl (P.K.-S.); r.djavadian@nencki.edu.pl (R.D.); 2 Faculty of Biology and Environmental Sciences, Cardinal Stefan Wyszynski University in Warsaw, 01-938 Warsaw, Poland

**Keywords:** axon guidance mechanisms, anterior commissure, Satb2, astrocytes, opossum, marsupial neurodevelopment

## Abstract

In marsupials, upper-layer cortical neurons derived from the progenitors of the subventricular zone of the lateral ventricle (SVZ) mature morphologically and send their axons to form interhemispheric connections through the anterior commissure. In contrast, eutherians have evolved a new extra callosal pathway, the corpus callosum, that interconnects both hemispheres. In this study, we aimed to examine neurogenesis during the formation of cortical upper layers, including their morphological maturation in a marsupial species, namely the opossum (*Monodelphis domestica*). Furthermore, we studied how the axons of upper layers neurons pass through the anterior commissure of the opossum, which connects neocortical areas. We showed that upper-layer II/III neurons were generated within at least seven days in the opossum neocortex. Surprisingly, these neurons expressed special AT-rich sequence binding protein 2 (Satb2) and neuropilin 1 interacting protein (Nrp1), which are proteins known to be essential for the formation of the corpus callosum in eutherians. This indicates that extrinsic, but not intrinsic, cues could be key players in guiding the axons of newly generated cortical neurons in the opossum. Although oligodendrocyte precursor cells were present in the neocortex and anterior commissure, newly generated upper-layer neurons sent unmyelinated axons to the anterior commissure. We also found numerous GFAP-expressing progenitor cells in both brain structures, the neocortex and the anterior commissure. However, at P12–P17 in the opossums, a small population of astrocytes was observed only in the midline area of the anterior commissure. We postulate that in the opossum, midline astrocytes allow neocortical axons to be guided to cross the midline, as this structure resembles the glial wedge required by fibers to cross the midline area of the corpus callosum in the rodent.

## 1. Introduction

Therian mammals, containing the modern lineages of marsupials and eutherians, diverged from each other at least 160 million years ago [1]. Eutherian mammals are a large infraclass of therians, comprising around 95% of all living mammals. Marsupials are divided into two groups, American marsupials (Ameridelphia) and Australian marsupials (Australidelphia), most of which are found in Australia. The main difference between the two therian infraclasses is in the time of birth in relation to the developmental phase: marsupials are born at a much earlier stage of development than eutherians [2].

Comparative studies of various marsupial and eutherian species have indicated that the sequence of neocortical development in all mammals is similar [3]. The discovery of the general homology of the developmental sequence of the cerebral cortex in all mammals leads to the conclusion that all this important evolutionary change took place at the beginning of mammalian radiation. Despite the similar six-layered cerebral cortex organization in both eutherians and marsupials, some essential differences exist. Although both eutherian and marsupial mammals show neocortical developmental gradients of the brain, the development of cerebral cortical layers in marsupials occurs after birth and takes longer than in eutherians [4,5,6]. The second main difference lies in the organization of interhemispheric connections. In the opossum, the lack of a corpus callosum causes the axons of neurons in the upper layers to turn toward the anterior commissure and then cross the midline to reach the contralateral hemisphere [7,8].

The connection of neocortical areas through the anterior commissure is 2–5 times longer (depending on the brain regions) than that in the corpus callosum, which slows down the process of the interhemispheric integration of sensory information and the coordination of the precise movements of all four extremities. Despite this, some marsupial species show a particular dexterity in their movements. For example, Australian marsupial mammals such as gliders and kangaroos move quickly over uneven terrain and rock cliffs [5]. Ameridelphia marsupial opossums also thrive in an environment where they face competition with eutherians. Nonetheless, these studies support the hypothesis that the central nervous system of eutherians has some advantages for more complex mental tasks.

Genetic factors govern the development of interhemispheric connections. Comparative studies of brain development in marsupials and eutherians provide evidence for understanding the changes that led to evolution at the genetic level. Several studies have documented the genes involved in the development of interhemispheric pathways in eutherian species. Research in mice has identified many genes that control the development of the corpus callosum [9]. Some proteins, transcription factors such as the erythropoietin-producing hepatocellular carcinoma cell-derived (Eph) family, and special AT-rich sequence binding protein 2 (Satb2) play an essential role during the development of axon projections [10,11,12]. Transgenic mice with a mutation in one of the EphA tyrosine kinase receptors, mainly the EphA5 receptor (EphA5(K-), with the intracellular kinase domain deleted), have shown that callosal axons of the somatosensory and visual cortices do not reach contralateral neocortical areas [11]. Neocortical axon guidance molecules such as Slit 2 or neuropilin 1 (Nrp1), a surface receptor for Sema3A, are also critical for the development of axon tracts [13,14]. However, such research on marsupial species is limited. Research related to the development of interhemispheric fiber tracts is also lacking. We hypothesized that Satb2 is not expressed in the upper cortical neurons of marsupials since their brains lack the corpus callosum. Identifying the molecular basis for the development of the corpus callosum in eutherians and the anterior commissure in marsupials allows us to understand the changes that lead to evolution.

The aim of the current study was to examine the neocortical neurogenesis and neuronal morphological maturation of these neurons. We asked whether astrocytes regulate the neuron axon pathways of neocortical interhemispheric connections in the development of opossums, *Monodelphis domestica*. Among the marsupials, the small, pouchless opossum has become a popular laboratory animal. This species is particularly well suited for developmental studies, and young opossums are the model of choice for ex utero studies of the mechanisms of early developmental events in the mammalian brain [15,16,17]. However, the Monodelphis opossum has more advantages as an animal model. The Monodelphis opossum has an omnivorous diet similar to that of humans. Therefore, it makes a better model for diet-induced research than laboratory rodents [18]. Our findings regarding the mechanisms that confine the growing cortical interhemispheric fibers to a different route than in the eutherians bring a deeper understanding of the mechanisms involved in neurodevelopment and mammalian evolution.

## 2. Results

### 2.1. BrdU-Labeled Cells in the Cerebral Cortex and Anterior Commissure

**Figure 1:**
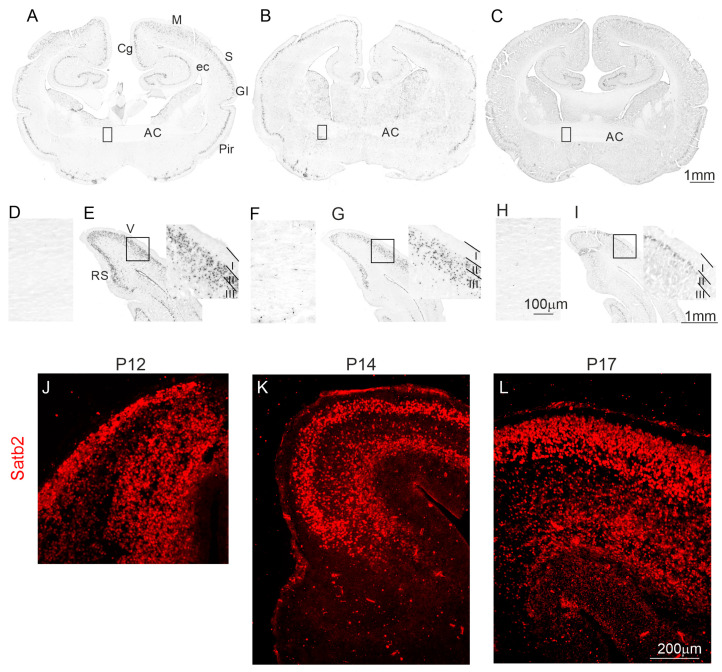
The distribution of BrdU+ and Satb2+ cells in the developing opossum brains. (**A**–**C**) coronal sections from the brain rostral part of P12 (**A**), P14 (**B**), and P17 (**C**), showing BrdU-labeled cells in the cingulate cortex, motor cortex, somatosensory cortex, granular insular cortex, piriform cortex, and anterior commissure. (**E**,**G**,**I**) coronal section from the brain caudal part of P12 (**E**), P14 (**G**), and P17 (**I**), demonstrating BrdU-labeled cells in the retrosplenial cortex and visual cortex. (**A**–**C**,**E**,**G**,**I**) BrdU-labeled cells were located in the neocortical upper layers of opossums that were injected at P12, P14, and P17, and sacrificed at P90. A few BrdU-immunopositive cells were observed in the anterior commissure at P12 (**A**,**D**), and their numbers gradually increased at P14 (**B**,**F**), and P17 (**C**,**H**); (**D**,**F**,**H**) Zoomed areas shown in (**A**), (**B**) and (**C**), respectively. (**J**–**L**) Immunostaining of developing cortical plate with Satb2 (red) at P12, P14, and P17 in opossum brains. The scale bar in (**C**), (**H**), and (**I**) refers to (**A**,**B**), (**D**,**F**), and (**E**,**G**), respectively. The scale bar in (**L**) refers to both images (**J**) and (**K**). AC, anterior commissure; Cg, cingulate cortex; ec, external capsule; GI, granular insular cortex; M, motor cortex; P, postnatal day; Pir, piriform cortex; RS, retrosplenial cortex; S, somatosensory cortex; V, visual cortex.

To study the neurogenesis of the cerebral cortex and anterior commissure, opossums of different ages were injected with bromodeoxyuridine (BrdU), a marker for DNA synthesis. Several studies reported that BrdU, when injected into pregnant laboratory rodents (mice or rats), can be toxic for embryos, inducing cell death in specific brain areas or producing a decrease in body and brain masses [19,20]. Additionally, in our previous study, all pups from each litter were injected on the same day [6]. We found that the upper-layer IV started to form at P12 [17]. Comparing the body masses of different litters of intact pups on a selected day, we found significant differences between the body masses of opossum pups from different litters on the same day of development. For example, at P17, the average mass of opossum pups from different litters varied by as much as 50% (0.97 ± 0.06 g vs. 1.6 ± 0.08 g). However, we were unable to weigh the opossum pups after the injection of BrdU because the pups touch their mother’s teat until they are four weeks old. During this period, removing the pup and touching the mother’s teat again is impossible. Therefore, here, opossum pups from each litter were injected on three different days (P12, P14, and P17), and BrdU-labeled cells were detected at P90. Our results showed that at P12, neurogenesis occurred in the upper layers of the cerebral cortex (Figure 1A,E). According to the cortical developmental gradients, visual areas developed later than the somatosensory or motor cortical areas placed in the posterior part of the brain (Figure 1A–C). At P12, the enhanced generation of visual neocortical neurons occurred in layers II–IV (Figure 1E), while the production of anterior neocortical neurons was observed only in layers II/III or II (Figure 1A). In particular, in the cingulate and somatosensory cortex, BrdU-labeled cells were located inclusively in the upper layer II (Figure 1A). This indicates that neurogenesis in the upper layers started one or two days earlier. At this age, BrdU-labeled cells were almost absent in the anterior commissure (Figure 1A,D). At P14, in the anterior part of the neocortex, neurons were mostly induced in layers II (Figure 1B); meanwhile, in the posterior part of the visual areas, BrdU-labeled cells were located in layers II–IV (Figure 1G). In the 17-day-old opossums, BrdU-labeled cells were detected only in the upper layer II of the visual area (Figure 1C,I). A few BrdU-labeled cells appeared in the anterior commissure at P12 (Figure 1A,D), and their number increased as the opossums developed (Figure 1B,C,F,H).

Next, we analyzed some markers, such as Satb2 and Nrp1; these are the receptors for semaphorin 3A and are known to be involved in the formation of the corpus callosum in rodents. Satb2 expression is found in the developing cerebral cortex of mice and is used as a layer-specific neuronal marker of the neocortex [21,22]. The dysregulation of the migration process and the misrouting of callosal axons was observed in mutant mice with Satb2 gene loss. Therefore, the axons of neocortical neurons reached the opposite hemisphere via the anterior commissure [23,24]. Nrp1 is also expressed in superficial layer neurons in mice [25], and the lack of Nrp1 receptors leads to the disorganization of axons in the corpus callosum [26]. We performed immunohistochemistry to study the presence of Satb2 and Nrp1 proteins in developing opossum brains. The use of specific markers revealed that upper-layer neurons of the opossum cerebral cortex were stained by Satb2 (Figure 1J–L). At P12 in the developing neocortex of the opossum, Satb2 was widely expressed in the upper layers (layers IV–III) neurons (Figure 1J). At P14 and P17,as the upper layers III-II were created, the number of Satb2-labeled cells increased (Figure 1K,L). Satb2+ cells were also visible in the deep layers of the neocortex. However, the intensity of Satb2 expression was lower than in the upper layers (Figure 1L).

**Figure 2:**
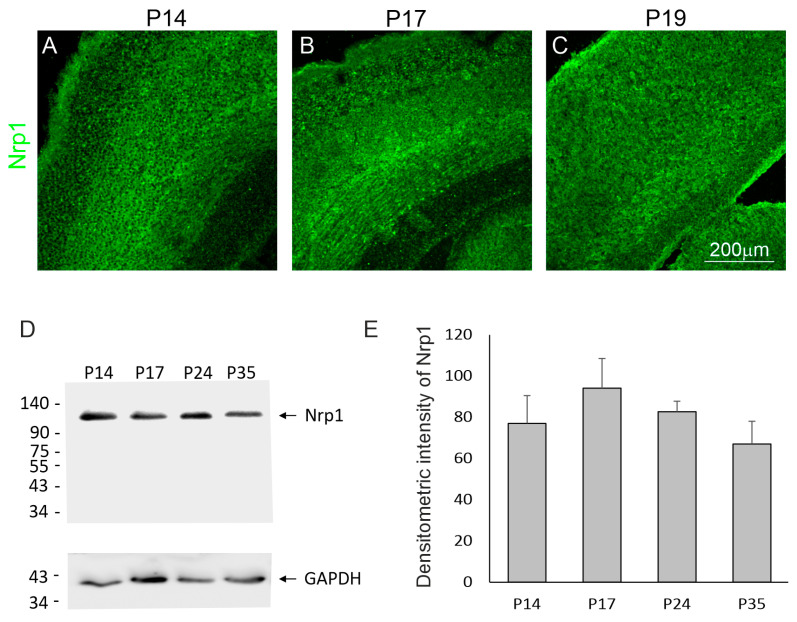
Nrp1 protein in the developing cerebral cortex of opossums. (**A**–**C**) The distribution of Nrp1-immunolabeled cells in the cerebral cortex of P14, P17, and P19 opossums. (**D**) Representative Western blot of Nrp1 and loading control GAPDH protein expression in the cerebral cortex of opossums at P14, P17, P24, and P35. (**E**) The quantification of Nrp1 protein in the developing cerebral cortex. The scale bar in (**C**) refers to both (**A**,**B**).

In contrast, deeper-layer neurons, particularly cells located in the intermediate and ventricular layers, were immunostained with antibodies against Nrp1 (Figure 2A–C). Western blot analysis of the Nrp1 quantification in the developing brain of the opossums detected that there were no differences in the amount of protein in the cerebral cortex (F_(3,12)_ = 1.138, *p* = 0.37) at the different developmental stages of opossums (Figure 2D,E). Together, these results suggest that intrinsic cues such as Satb2 and Nrp1 are not decision-making genes critical for axon guidance and interhemispheric connections.

### 2.2. Cellular Organization of the Anterior Commissure

**Figure 3:**
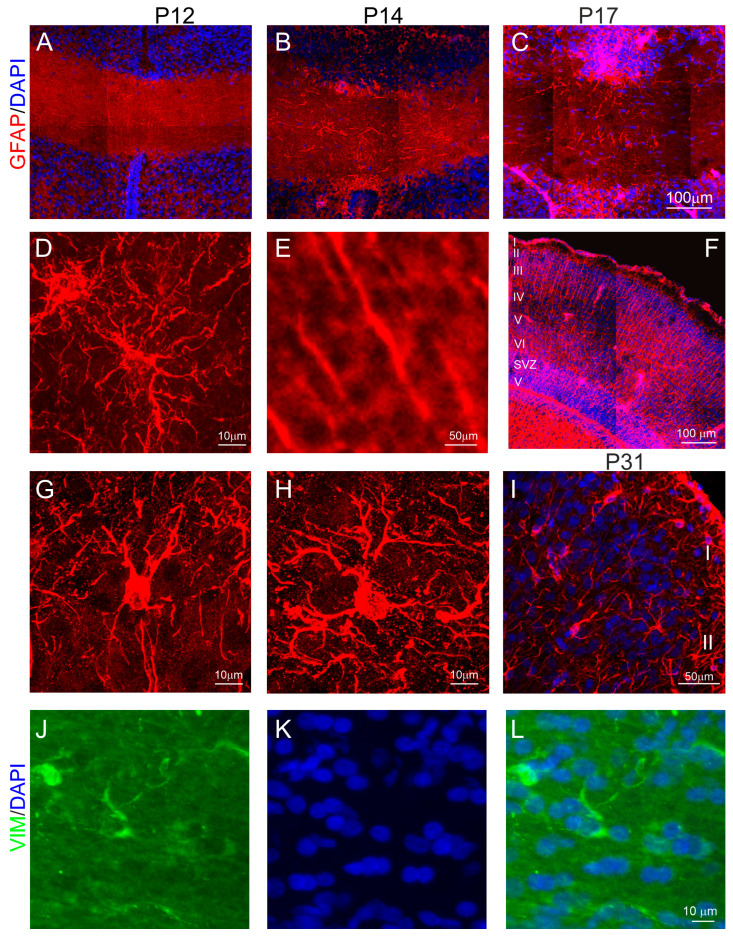
Development and cellular organization of the anterior commissure. (**A**–**C**) Double GFAP (red) and DAPI (blue)-immunolabeled brain sections from the anterior commissure at P12 (**A**), P14 (**B**) and P17 (**C**), and the cerebral cortex (**F**,**I**) at P17 (**F**) and P31 (**I**) in the opossums. (**D**,**E**,**G**,**H**) High magnification confocal images showing GFAP-labeled cells in the anterior commissure of P17 (**D**) and cerebral cortex of opossums at P17 (**E**) and P31 (**G**,**H**). (**L**) Double labeled with DAPI (**K**) and vimentin (**J**)-stained brain coronal sections at the level of the anterior commissure. The scale bar in (**C**), and (**L**) refers to (**A**,**B**), and (**J**,**K**), respectively.

In marsupials, the formation of the anterior commissure is linked to the generation of neocortical neurons, whose axons project into the anterior commissure and cross its midline region to form interhemispheric connections. We found that the anterior commissure connecting the brain’s two cerebral hemispheres was visualized in DAPI-stained brain sections at P12 in the opossums (Figure 3A). DAPI is a marker for the nucleus of all types of cells. This structure gradually became thicker with brain development, as shown in a 14-day-old (Figure 3B) and a 17-day-old opossum (Figure 3C). During this developmental period, cells of the midline region of the anterior commissure expressed GFAP. The morphology of some GFAP-labeled cells indicated that these were not radial glial cells (Figure 3A–C). Consistent with other studies [27,28], we observed typical radial glial cells in the cortex (Figure 3E,F), which were characterized by long radial glial processes and their somata being placed in the ventricular zone (Figure 3F). These cells were progenitor cells that gave rise to neurons and glial cells. In the cerebral cortex, astrogliogenesis occurs later, mainly at the end of the formation of cortical layers [29]. There are two types of astrocytes, namely protoplasmic and fibrous. Protoplasmic astrocytes have short, thick, and more branch processes, while fibrous astrocytes have long, thin, and a few processes [30]. We found both protoplasmic and fibrous astrocytes in the cerebral cortex at P31 in the opossums (Figure 3G,H). Interestingly, we detected astrocytes that were GFAP+ in the midline of the anterior commissure of the developing opossum (Figure 3D). Additional immunostaining was performed using vimentin, a commonly used astrocyte marker. Our results revealed that vimentin-positive cells were present in the midline region of the anterior commissure (Figure 3J–L).

**Figure 4:**
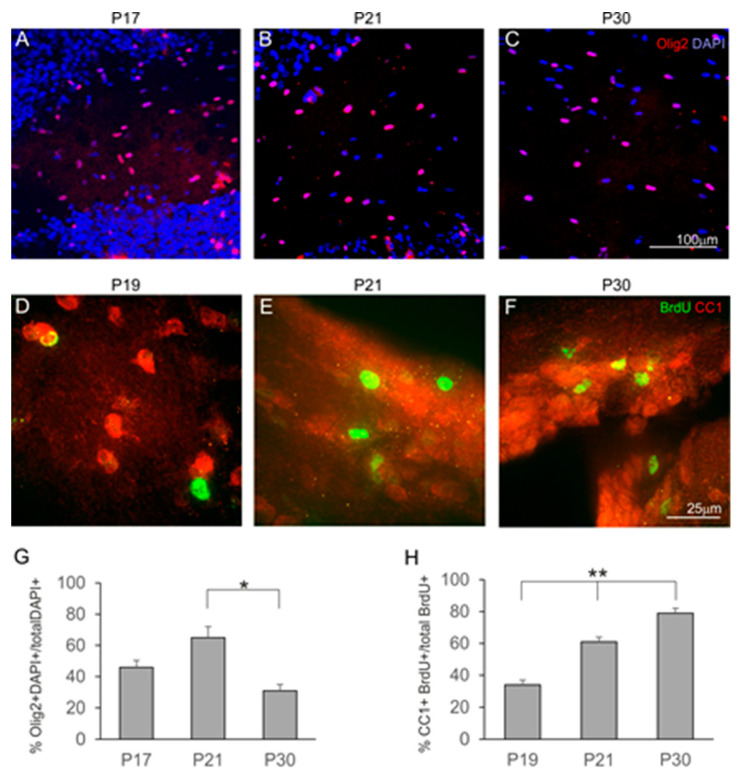
Development of oligodendrocytes in opossums. (**A**–**C**) Olig2 (red) and DAPI (blue) immunostaining in the anterior commissure of opossums at P17 (**A**), P21 (**B**) and P30 (**C**). (**D**–**F**) BrdU (green) and CC1 (red) immunolabeled cells in brain sections presenting the anterior commissure in opossums at P19 (**D**), P21 (**E**) and P30 (**F**). (**G**,**H**) Percentage of cells colocalizing Olig2 and DAPI (**G**) or BrdU and CC1 (**H**) expressed as mean ± SEM. * *p* = 0.02, ** *p* < 0.0001. The scale bar in (**C**), and (**F**) refers to (**A**,**B**), and (**D**,**E**), respectively.

To determine the cellular organization of the anterior commissure, brain sections of opossums at various developmental stages stained with different cellular markers were analyzed. We found a few DAPI staining cells in 12-day-old opossum brains, whose numbers increased with the opossum age (Figure 4). To identify cell types, double immunostaining was carried out with DAPI and Olig2, a marker for both oligodendrocyte precursor cells and mature oligodendrocytes, as well as astrocytes. We showed that a number of DAPI-stained cells were colocalized with Olig2 (Figure 4A–C), indicating that these were progenitor cells for oligodendrocytes or astrocytes. At P17, 46% of DAPI-stained cells were colocalized with Olig2, and their number increased to 65% in the 21-day-old opossums (Figure 4A,B). A one-way ANOVA test revealed that there was a significant difference between the double-labeled (DAPI and Olig2) cells (F_(2,10)_ = 5.5, *p* = 0.02) during development (Figure 4G). Mature oligodendrocytes were developed later. Double-labeling for BrdU and CC1, a marker for mature oligodendrocytes, revealed that at P19, only 34% of cells were oligodendrocytes, while their number was almost doubled (61%) by the age of 21 days (Figure 4D,E,H). The highest number of double-labeled cells (79%) was observed at P30 (Figure 4F,H). Statistical analysis showed that the number of double-labeled cells significantly increased during development (F_(2,14)_ = 29.25, *p* < 0.0001).

**Figure 5:**
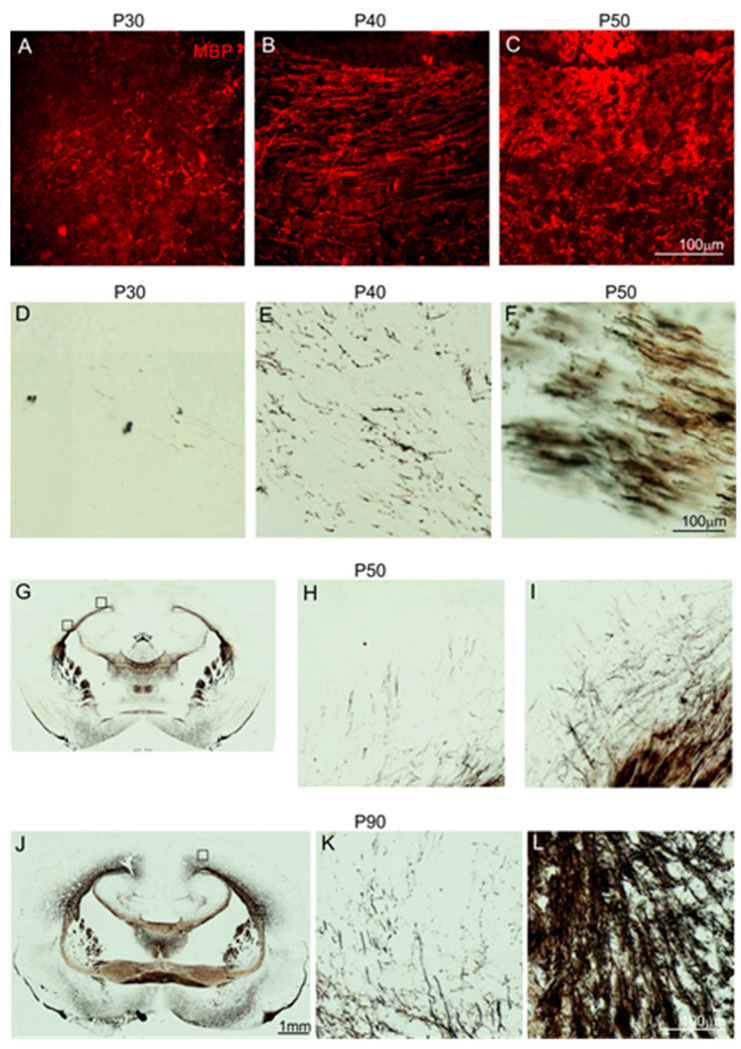
Myelination of the anterior commissure in the developing opossum. (**A**–**C**) Myelin basic protein (MBP) immunostaining in the anterior commissure of opossums at P30 (**A**), P40 (**B**) and P50 (**C**). (**D**–**L**) Fiber staining of the anterior commissure using the Gallyas silver impregnation method. Individual myelinated axons were visualized in the anterior commissure in the 30-day-old opossum brain (**D**), and the intense staining of fiber tracts was seen in 50-day-old (**F**–**I**) and 90-day-old opossums (**J**–**L**). The scale bar in (**C**) refers to both (**A**) and (**B**). The scale bar in (**F**) and (**J)** refers to (**D**,**E**,**H**,**I**,**K**,**L**), and (**G**), respectively.

Mature oligodendrocytes form myelin sheaths on neuronal axons that enable an increase in the speed of electrical conduction. To examine the myelination process of axons in the anterior commissure, immunostaining was applied for myelin basic protein (MBP). MBP is the main protein produced by oligodendrocytes for the organization of myelin sheaths. This protein appeared at low levels in the anterior commissure of 1-month-old opossums (Figure 5A). The intensity of the MBP labeling increased as development progressed (Figure 5A–C). In 50-day-old opossums, abundant MBP+ structures were detected in the anterior commissure (Figure 5C).

These data were verified using another histological technique based on the argyrophilic property of myelin. Myelin has an affinity for silver and binds silver particles. This method, also called Gallyas silver impregnation for the visualization of myelin fibers, confirmed the data obtained by the MBP evaluation. We detected that single myelinated axons were present in the anterior commissure of 30-day-old and 40-day-old opossums (Figure 5D,E). Like MBP, the intensity of myelin staining using the Gallyas method increased with age. In 50-day-old and older opossums, numerous myelinated fibers were observed in the anterior commissure (Figure 5F–L).

The opossum, Monodelphis domestica, is a pouchless marsupial. After birth, newborn opossums attach to their mother’s teats, causing the teat to swell, and remain attached to their mother for up to 4 weeks. One-month-old opossums detach from their mother’s teats and start learning their environment, which is associated with the development of the senses. We observed that the myelination process starts at P30 in opossums and is linked with the development of the senses. The number of myelinated axons increases as more senses develop. For example, at P40 in opossums, an increased number of myelinated axons in the anterior commissure is associated with the development of vision as the opossums open their eyes at P35–P37. During weaning in 2-month-old opossums, myelinated axons remarkably increase in the anterior commissure and the cerebral cortex. Taken together, these data demonstrate that the myelination of the neuronal axons is linked with the development of senses that allow opossums to learn their environment.

### 2.3. The Pattern of Neocortical Axons Forming the Anterior Commissure

**Figure 6:**
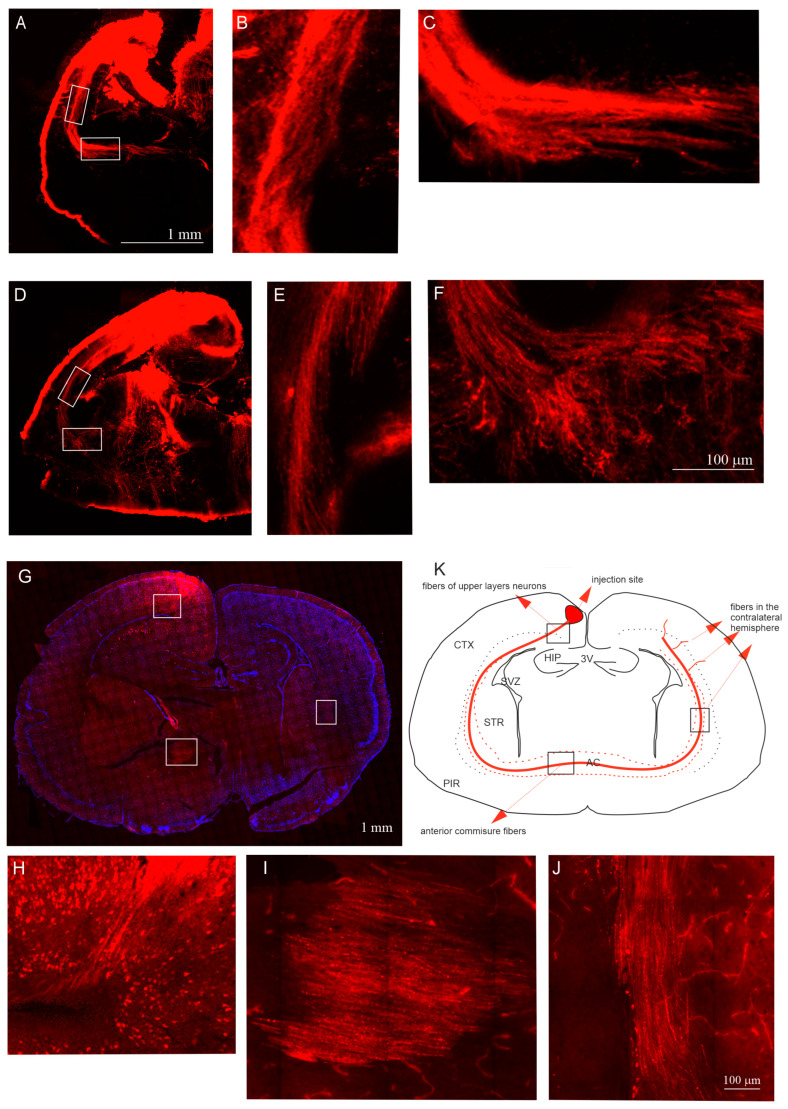
The pattern of axon projection neocortical neurons in the developing opossum brain is defined by DiI labeling (**A**–**F**) or virus infection (**G**–**J**). The pattern of the DiI-labeled axons of neocortical neurons in 12-day-old (**A**–**C**), 14-day-old (**D**–**F**), and 30-day-old (**G**–**J**) opossum brains. (**B**,**E**) and (**C**,**F**) The zoomed area showing the external capsule (**B**,**E**) and the anterior commissure (**C**,**F**) in 12-day-old (**B**,**C**) and 14-day-old (**E**,**F**) opossums. (**H**) the zoomed area from the coronal brain section (**G**), showing just below the injection site; (**I**) the zoomed area from the anterior cortex, showing mCherry-expressing axons; and (**J**) the zoomed area from the contralateral neocortex. (**K**) Schematic representation of the coronal section presented in G, showing the injection site and fiber tract containing neocortical neuronal axons that cross the anterior commissure and reach the contralateral neocortex. The scale bar in (**A**), and (**F**) refers to (**D**), and (**B**,**C**,**E**), respectively. The scale bar in (**J**) refers to both images (**H**,**I**). 3V, third ventricle; AC, anterior commissure; CTX, cerebral cortex; HIP, hippocampus; PIR, piriform cortex; STR, striatum.

We conducted ex vivo experiments to study the neocortical axonal pathways during development in the opossum. The brains of opossums of different ages were isolated and subsequently evaluated with a DiI tracer. DiI is a fluorescent lipophilic dye that results in the anterograde and retrograde labeling of neurons. DiI diffusion along long axon membranes lasted for several weeks. Therefore, the brains of opossums injected with DiI were incubated in 4% paraformaldehyde for 6–8 weeks. The injection sites of DiI in 12- and 14-day-old opossums were large and extended into all cortical layers. Moreover, the DiI injections spanned into the hippocampus. Although the injection site was too large, tracing the axons of the upper-layer neurons and tracking the axon pathways were still possible. We observed that interhemispheric cortical connections are widely distributed during these developmental stages. Injections of DiI into the cortex of 12-day-old (Figure 6A–C) and 14-day-old (Figure 6D–F) opossums revealed that the pattern of DiI labeling is similar in the brains of both age groups. We demonstrated that neocortical neurons send their axons to the white matter of the neocortex, forming an external capsule, and then the axons reach the anterior commissure (Figure 6A–F).

Our findings revealed that the method employed when using the DiI tracer in opossum pups to examine the axon pathways from a small brain area has some limitations. Opossum brains at P12 and P14 are too small to target just the pyramidal neurons in the neocortex’s superficial layers II/III. Even small injections of DiI or DiI crystals into the neocortex’s upper layers spread through the cortex to subcortical structures.

To achieve precise injections of the tracer into the upper cortical layers of opossums’ neocortex, a virus was injected into the upper cortical layers of 30-day-old animal brains. We applied a virus carrying information about the mCherry red fluorescent protein expressed in excitatory cells, showing the presence of CaMKII (Ca^2+^/calmodulin complex kinase). The virus injections revealed that the axons of mCherry-expressing neurons from the upper layers II/III were visible just below the deeper cortical layers, along the surface of the thalamus (Figure 6G,H). These axons reached homotopic and heterotopic neocortical areas of the contralateral hemisphere through the anterior commissure (Figure 6G,I,J).

## 3. Discussion

This study demonstrated the birthdate of upper-layer neurons in the opossum neocortex, whose axons project through the anterior commissure to the contralateral neocortex. We found that the generation of neocortical neurons in the upper layers II/III lasts more than six days. Newly generated cortical neurons of the upper layers expressed Sabt2 and Nrp1 proteins, which are critical for the formation of the corpus callosum in eutherians. Satb2 was mainly expressed in the upper-layer neurons, while Nrp1 was expressed in the deeper-layers neurons. A population of GFAP-expressing cells was detected in the midline region of the anterior commissure. The further identification of these cells showed that they had the morphology of astrocytes. Neocortical neuronal tracing with DiI or a virus showed that upper-layer neurons send their axons to the white matter of the neocortex, forming an external capsule. Then, these axons crossing the midline of the anterior commissure travel to the contralateral cortex. Our results also revealed that oligodendrocytes progenitors were present in the cortex and anterior commissure of 14-day-old opossum brains, but that the myelination of cortico-cortical fibers started later. The first myelinated axons appeared in the anterior commissure in 30-day-old opossums, which is associated with the development of the senses.

The axons of the upper-layer pyramidal neurons, which form long interhemispheric connections, allow communication between the two hemispheres. A time course analysis using BrdU, which determines cell proliferation, has shown that neocortical development in opossums is protracted compared to that in mice [6,15,16,31]. Several papers have reported that BrdU is toxic for embryos, producing cell death and decreasing cell proliferation [19,20]. Further papers have shown that the toxicity of BrdU is associated with high or repeated doses. The teratological effects of BrdU on the body and brain have also been reported [19]. We used a minimal, single dose of 20 mg/kg BrdU for the injection into opossums. Unfortunately, we cannot weigh opossum pups when they hang on their mother’s teats during the first month. We weighed the BrdU-injected opossums at the age of three months before they were sacrificed. Their body and brain weights did not differ from those of intact animals at the same age.

To study the generation of cortical upper-layer neurons, opossum pups from the same litter were used to note differences in the body masses of the intact opossum pups between different litters on the same developmental day. In our previous paper, we found that the formation of upper layers IV/III started at P12. Therefore, opossum pups from each litter were divided into three groups, and each group of pups was injected with BrdU at 12, 14, or 17 days of age, respectively. Our data on the BrdU labeling of cortical cells in developing opossum brains demonstrated that cortical gradients, particularly a rostroventral to the caudodorsal gradient in the opossum neocortex, are apparently distinguished. Due to the presence of cortical gradients, we could notice that neurons of the anterior cortex from upper-layer III in the opossum were generated a day or two earlier, but not at P12 as we had assumed. We further suggest that the development of the cerebral cortex is slightly delayed in low-weight opossum pups of the same age. Our finding also demonstrated the advantages of using opossums as an animal model in neurodevelopmental neuroscience compared to laboratory rodents. Mice need six days to develop all six neocortical layers, while in opossums, only the upper layers form in at least seven days. Due to the intense and short period of neurogenesis that occurs during the formation of the neocortex in mice, cortical developmental neurogenic gradients are not clearly observed [31]. Therefore, it is not easy to notice subtle developmental differences in the neocortex of mice.

In marsupials, neocortical axons project to the anterior commissure, which becomes the main route providing connection between the cerebral hemispheres [32,33,34]. Numerous neuron axons (55%) projecting to the anterior commissure were placed in layer III, but only 12% were placed in layer V [35]. Meanwhile, in eutherians, neocortical interhemispheric connections are formed mainly by axonal fiber bundles passing through the corpus callosum [36,37]. This structure is formed by projections of cortical neurons localized in both upper layers II/III and layer V. There are many genes that control the specificity of neocortical upper-layer cells and their projections. The absence of the transcription factors Pax6 or Tlx has been shown to reduce the number of upper-layer neurons generated and disorganize commissural connections [38].

In the developing opossum neocortex, we analyzed the localization of proteins, which are known to play a critical role in the development of the mouse corpus callosum. For example, the Nrp1 receptor is crucial for the formation of the corpus callosum of rodents. In transgenic Npn1Sema mice, the Npn1 receptor is not able to bind its ligand. The cingulate cortex of these mice is decreased in size, and the corpus callosum in the rostral part is absent. Although the Nrp1 receptor is associated with the corpus callosum, we showed that this protein is located in neurons of the opossum neocortex. We found that the level of the Nrp1 receptor changes during the development of the opossum’s neocortex. The highest level of this protein was recorded during the period in which upper layers II/III were forming in 14-day-old opossums (P14). In the following days of opossum development, the level of Nrp1 was decreased in the neocortex.

We have also studied the expression of Sabt2, a DNA-binding protein in the neocortical neurons of opossums; this is a key player that affects the formation of the corpus callosum in laboratory rodents. Satb2 expression was observed in the neocortex at embryonic day (E) 13 in the mouse and E16 in the rat [23,24]. Our data have demonstrated that in the developing opossum neocortex, Satb2 is expressed in upper-layer neurons. At P12–P17 in the opossums, the number of Satb2+ neurons in the neocortex gradually increased as upper layers III-II were formed. This pattern of Satb2-expressing cells is similar in the neocortex of mice. It was suggested that Satb2 is involved in regulating gene expression through interaction with the chromatin structure and by binding to DNA sequences in differentiating cortical neurons [39,40]. Satb2 is predominantly expressed in the upper-layer neurons of the rodent neocortex. The insertion of a LacZ gene in the Satb2 locus of mutant mice resulted in the dysregulation of the migration process [23]. At E18.5, these mice with Satb2 gene loss showed thinner cortices due to neuronal migration defects. Satb2 binds to the regulatory elements of Ctip2 and acts as a repressor. In addition, Satb2-deficient neocortical neurons reorganized their projections. Their axons did not reach the corpus callosum, but they projected to subcortical structures through the internal capsule. An analysis of Satb2 knock-out mice brains demonstrated that the cortical axon connections are reorganized due to inadequate gene expressions. Instead of cortico-cortical connections, some upper-layer cells produced cortico-subcortical or cortico-spinal connections. The corpus callosum was not formed in Satb2 knock-out mice, and the axons of neocortical neurons reached the opposite hemisphere via the anterior commissure [24]. Surprisingly, our research showed that the expression of the Satb2 protein in the opossum cortex is very similar to that of mice. Our results indicate that the differences in the genetic programs that determine a different direction of axon growth in upper-layer cells in the opossum cortex are not associated with Satb2. This protein may have different effects on the cooperating proteins within different species that are regulated by environmental signals, and axons are guided to reach their target mainly by extrinsic cues.

In eutherians, the formation of interhemispheric neocortical connections occurs during embryonal development. The first axons that cross the midline above the hippocampal commissure are the axons of cingulate pioneer neurons, followed by neocortical axons, which cross as early as E16–E17 in mice [41,42,43]. Two days later, the growth of the corpus callosum progresses rapidly. In rats, the commissures continue to develop prenatally and postnatally, reaching adult size at P7 or a few days prior to myelination [44,45]. A similar developmental pattern of the corpus callosum has been observed in other eutherian species [46,47]. By contrast, the formation of fiber tracts in marsupials occurs postnatally. In the wallaby, the anterior commissure axons cross the midline at P14, and its development lasts till P80 [48,49]; meanwhile, in the Dydelphis opossum, neocortical axons start to grow at P12 and reach the contralateral cortex by approximately P35 [34]. Our data showed that in the opossum, *Monodelphis domestica*, interhemispheric neocortical connections were visible as early as P12 and at P14. Taken together, in both marsupials and eutherians, the developmental sequences of neocortical fiber tracts are similar, with the main exception being that neocortical axons project to the anterior commissure in marsupials and the corpus callosum in eutherians.

In general, both astrogenesis and oligodendrogenesis occur in the later stages of cerebral cortex development. In mice, neurons are generated within E12–E18, and only after the completion of neurogenesis, the first astrocytes appear during the late embryonic stage. The number of astrocytes increases in the following three weeks during postnatal development [50]. Most astrocyte precursors are produced by radial glia located in both the SVZ and ventricular zone of the developing brain [51]. These radial glial cells, after becoming glial-lineage-like cells, differentiate into astrocytes. Astrocytes are a heterogeneous population of cells. In rodents, the presence of two basic astrocyte subpopulations has been detected during cerebral cortex development, with the protoplasmic astrocytes mostly being placed in the gray matter and fibrous astrocytes being spread throughout the white matter [29]. In marsupials, protoplasmic and fibrous astrocytes develop in the neocortex after the cortical layers have completely formed. Our data demonstrated that in the cerebral cortex of 31-day-old opossums, the morphology of astrocytes was similar to that of mice [52]. Olig2, which is a transcription factor involved in the differentiation of oligodendrocytes and neurons, plays a crucial role in the specification and differentiation of astrocytes in cerebral white matter during development [53,54,55]. A lineage analysis of Olig2-expressing cells using a tamoxifen (TAM)-inducible Cre/loxP system showed that Olig2+ progenitors give rise to astrocytes at a late embryonic stage. We found that Olig2+ precursors were localized in the developing opossum anterior commissure. These results allow us to suggest that, during the development of the anterior commissure, Olig2+ precursors may be involved in the differentiation of astrocytes as, in the early stages of the formation of neocortical interhemispheric connections, mature oligodendrocytes are not developed. Understanding the differences and similarities in the development of astrocytes across various species of mammals can offer insight into astrocyte evolution. However, a lack of data on marsupials at this stage does not allow conclusions to be draw.

Although interhemispheric connections arise during the development of the opossum neocortex, the myelination process of axons in cortical neurons establishing their long connections, especially axons in the anterior commissure, starts later. The myelin sheath is a structure on neuronal axons that increases the speed of electrical conduction. The expression of MBP in the anterior commissure increased with development, reaching high levels in 50-day-old opossums. Based on our data, we suggest that the myelination of the neuronal axons is linked with the development of the senses that allow opossums to learn their environment. The same pattern observed in the myelination of the main fiber tracts has been observed in eutherians. The generation of oligodendrocytes occurs during embryonal development and continues into old age [56].

Several papers have reported that specific midline glial structures are essential for the guidance of commissural axons [57,58]. In particular, a glial wedge located bilaterally between the rostromedial cortex and septum directs the growth of neocortical callosal axons to reach their target, the corpus callosum. Glial wedge cells are not radial glial cells; they are specialized astrocytes that express GFAP. This population of GFAP+ glial cells appears earlier at mid-neurogenesis, between E13 to E17, and forms the glial wedge [58,59]. Bignami et al. [60], using GFAP immunostaining, were the first to describe midline glial structures. Later, a structure in the medial wall of the lateral ventricle was renamed by Shu and Richards [61] as the glial wedge. Glial wedge cells are generated in the ventricular zone of the lateral ventricle, and their cell bodies remain there, but radial–glial-like long processes are sent to the midline [51]. Glial wedge defects cause the agenesis of the corpus callosum [62]. We demonstrated that a population of glial cells is present in the midline of the anterior commissure in opossums. We suggest that these glial cells act as a substrate for fibers crossing the midline, like a glial wedge in eutherians, and this is most likely the reason why marsupials do not form a glial wedge.

Another mechanism for corpus callosum development in mice is associated with the pioneering cortical axons that reach and pass through the midline to form interhemispheric connections [42,63]. The axons of cingulate cortical pioneer neurons cross the midline, forming a path for subsequent crossing axons. Pioneer axons interact with several cues, including glial structures, while later axons interact with pioneer axons. In the Monodelphis opossum, the first fibers of the olfactory bulb neurons reach the midline of the anterior commissure between P7 and P10 [33]. Thus, the axons of olfactory bulb neurons are considered to be pioneer axons for neocortical axons. A few days later, projecting neocortical axons use GFAP+ astrocytes as a substrate to cross the midline of the anterior commissure.

There are some limitations to our study. First, during the development of upper-layer neurons, opossum brains are too small to target only the neocortical pyramidal neurons of the superficial layers of the neocortex. The injection of the tracer spreads through the cortex to subcortical structures. The labeling of axons crossing the anterior commissure is not affected. However, it is impossible to define whether the cortical area is visual or somatosensory. Second, we are unable to identify differences in the axon guidance molecules between marsupials and eutherians. Future research in this direction may help to develop methods for assessing the risk of occurrence and diagnosing developmental disorders of the formation of the corpus callosum in humans.

Taken together, knowledge of the developmental events that occur in the neocortex, including molecular signatures, may shed light on different developmental mechanisms that are essential for understanding neurogenesis and how guidance molecules define a direction of growth axons.

## 4. Materials and Methods

### 4.1. Animals

Opossums of both sexes bred at the Nencki Institute of Experimental Biology colony were used for this study. The housing facility was constantly monitored; the temperature was kept at 26–28 °C, the humidity was 50–70%, and the daily cycle was 14/10 h (day/night). Every effort was made to minimize the number of animals used and the level of stress they would endure. The study was carried out in compliance with the ARRIVE guidelines. The experimental procedures complied with the Polish Law on Experiments on Animals, which implements the European Council Directive, and were approved by the 1st Local Ethical Committee for Animal Experimentation in Warsaw (Permit Number: 486/2017 and 272/2017).

### 4.2. Injections of BrdU, DiI and Viruses

Opossums with pups attached to their nipples were anesthetized with 5% Isoflurane (Baxter, Warsaw, Poland). Then, three animals for each age group at P12, P14, P17 per litter, and opossums at P19, P21 and P30 (three animals for each age group), were injected subcutaneously with a single dose of 20 mg/kg BrdU (Sigma-Aldrich, St. Louis, MO, USA). Opossums at P90 were sacrificed by an overdose of pentobarbital (Morbital, Biowet, Pulawy, Poland) and perfused with saline followed by 4% paraformaldehyde (PFA). Their brains were removed, postfixed in the 4% PFA, incubated with 30% sucrose and cut into 40 μm coronal sections in a cryostat (Leica Biosystems, CM1860, Nussloch, Germany). The brain sections were arranged in a series of ten.

Opossums at P12 (n = 3) and P14 (n = 3) were anesthetized by hypothermia and decapitated. Their brains were isolated from the scull and a 2 mg/mL ethanol solution of 1,10-dioctadecyl-3,3,30,30-tetramethylindocarbocyanine perchlorate (DiI, Molecular Probes, Leiden, The Netherlands) was injected into the neocortex using a glass capillary, or the placement of DiI crystals was applied. The brains were incubated in 4% paraformaldehyde (PFA) in 0.1 M phosphate buffer (pH 7.4) for 6–8 weeks. The brains were then immersed in 30% sucrose in phosphate-buffered saline (PBS) and cut into 40 or 50 μm coronal sections in a cryostat.

Opossums at P30 or P31 (n = 3) were anesthetized with isoflurane and treated with painkillers (Lignocainum Jelfa 2%, PharmaSwiss, Prague, The Czech Republic). AAV1/2:CaMKII-mCherry virus with viral titer 1–2 × 106 genome copies/mL was injected with a glass capillary at a volume of 2 µL into the upper neocortical layers through the cartilaginous skull, which was exposed by a small skin incision. Brains were removed 35 days after injection and fixed in 4% PFA, incubated with 30% sucrose, and cut into 40 μm thick coronal sections.

### 4.3. Brain Tissue Preparation

Opossums at P12, P14, P17, P19, P21 were anesthetized by hypothermia and decapitated. Their brains were removed and fixed in a 4% PFA solution for a week. Opossums at P30, P40, P50 and P90 (3 to 5 opossums in each developmental age) were sacrificed by an overdose of pentobarbital and perfused with saline followed by 4% PFA. Their brains were then cryoprotected with a 30% sucrose solution and cut in a cryostat (Leica Biosystems, CM1860, Nussloch, Germany) into 40 μm coronal sections (free-floating sections collected in an antifreeze solution) or into 20 μm sections on glass slides. Brain sections were arranged in a series of ten and stored at −20 °C until use.

### 4.4. Myelin Staining by Gallyas Silver Impregnation

The brain sections of the anterior commissure collected from animals at P30, P40, P50, P90 were stained for myelinated fibers visualization. The sections on glass slides were placed in a solution of pyridine (POCh, Gliwice, Poland) with acetic anhydride (Fisher Scientific GmbH, Schwerte, Germany) (2:1) for 30 min. The sections were then rinsed with water and placed in 0.1% silver nitrate (Fisher Scientific GmbH, Schwerte, Germany) and 0.1% ammonium nitrate solution (POCh, Gliwice, Poland) with a pH of 7.5 for 45 min. After rinsing with 0.5% acetic acid solution (POCh, Gliwice, Poland), the sections were placed in the developer solution for 5 min. The developer consisted of three different solutions containing anhydrous sodium carbonate, silver nitrate, ammonium nitrate, tungstic silicic acid (Sigma-Aldrich, Darmstadt, Germany) and formalin (POCh, Gliwice, Poland). Subsequently, sections were rinsed in 1% acetic acid solution and then placed in 0.2% potassium ferricide solution (POCh, Gliwice, Poland) for 5 min. The last steps, incubation in developer and washing, were repeated several times. Then, the brain sections were rinsed in a solution of 0.5% sodium thiosulfate (POCh, Gliwice, Poland), dehydrated in three different ethyl alcohol solutions (50%, 70% and 98%), degreased in xylene and covered with DePeX (Serva, Heidelberg, Germany).

### 4.5. Immunofluorescent Labeling

The immunofluorescent labeling of opossum brains from P12 to P21 was performed on glass slides with brain sections, while at P30 to P50, free-floating brain sections were used (n = 5 for each group). The brain sections were incubated for 1 h with either 10% normal goat serum, 10% normal chicken serum (Sigma-Aldrich), or 1% bovine serum albumin (BSA) in PBS. Next, the sections were incubated overnight with primary antibodies: mouse anti-Olig2 (1:100, Millipore, Kankakee, IL, USA), rabbit anti-GFAP (1:500, Dako, Dublin, Ireland), mouse anti-vimentin (1:500, Sigma-Aldrich, St. Louis, MO, USA) goat anti-MBP (1:100, Santa Cruz Biotechnology, Heidelberg, Germany), rabbit anti-Satb2 (1:200, gift from Tarabykin lab, Berlin, Germany), and rabbit anti-Nrp1 (1:200, Abcam Cambridge, UK). Appropriate secondary antibodies, namely goat anti-rabbit 568, chicken anti-goat 568, goat anti-mouse 568, goat anti rabbit 488 (all 1:500, AlexaFluor Invitrogene, Waltham, MA, USA) and goat anti-rat biotinylated antibody (1:200, Jackson ImmunoResearch, Cambridgeshire, UK), followed by streptavidin conjugated with fluorochrome 488 (1:500, AlexaFluor Invitrogene, Waltham, MA, USA), were used. Then, the brain sections, after rinsing with PBS, were counterstained with DAPI (1:5000, Sigma-Aldrich, Darmstadt, Germany). Finally, the brain sections were mounted on glass slides and coverslipped with 60% glycerol in PBS.

### 4.6. Immunolabeling for BrdU

The brain sections of the BrdU-injected opossums (P12, P14, P17 per litter) were washed with Tris–buffered saline (TBS). After denaturation in 2 M HCl at 37 °C for 30 min and washing with boric acid, endogenous peroxidases were blocked using 3% H_2_O_2_ in TBS. The brain sections were then rinsed in TBS with 0.1% Triton X-100 (TBS-A), and TBS-A with 0.05% bovine serum albumin (TBS-B). After blocking for 1 h in a 10% NGS (Gibco, Carlsbad, CA, USA) in TBS-B, the brain sections were incubated with rat anti-BrdU monoclonal primary antibody (1:500, Abcam, Cambridge, UK) overnight at 4 °C. After a 15 min wash with TBS-A and TBS-B, the brain sections were incubated for 60 min in biotinylated goat secondary antibody (1:200, Jackson ImmunoResearch, Cambridgeshire, UK) in TBS-B. This was followed by washes with TBS-A and TBS-B with horseradish peroxidase-conjugated extravidine (1:200, Sigma-Aldrich, St. Louis, MO, USA) for 1 h. The brain sections were rinsed in Tris buffer and stained with diaminobenzidine (DAB) substrate enhanced with nickel salts (DAB Substrate Kit, Vector Laboratories, Newark, CA, USA). The brain sections were spread onto glass slides, dried and coverslipped with DePeX.

#### Double Immunolabeling

One series of ten brain sections of animals injected with BrdU at P19, P21 and P30 were subjected to double/triple immunostaining. After denaturation and blocking as described above, the sections were incubated overnight in the mouse anti-CC1 (APC) (1:100, Abcam, Cambridge, UK) and rat anti-BrdU (1:500, Abcam, Cambridge, UK) primary antibodies. Appropriate secondary antibodies, namely goat anti-rabbit 568 (1:500, Abcam, Cambridge, UK) and biotinylated goat anti-rat secondary antibody (1:200, Jackson ImmunoResearch, Cambridgeshire, UK), followed by Alexa Fluor 488-conjugated streptavidin (1:500, AlexaFluor Invitrogene, Waltham, MA, USA), were subsequently used. Next, the brain sections, after rinsing with PBS, were counterstained with DAPI (1:5000, Sigma-Aldrich, Darmstadt, Germany). Finally, the brain sections were mounted on glass slides and coverslipped with 60% glycerol in PBS.

### 4.7. Western Blot

The brains from opossums at P14, P17, P24 and P35 (n = 3) were rapidly isolated, and the cerebral cortex was separated on ice and weighed. The tissue samples were mechanically homogenized in lysis buffer containing protease inhibitors (Roche, Basel, Switzerland), treated with NP40 detergents (Fluka, Buchs, Switzerland) and sodium dodecyl sulfate (SDS, Sigma-Aldrich, Darmstadt, Germany), and incubated for 15 min. Next, they were centrifuged at 14,000 rpm for 45 min at 4 °C. The supernatant was collected, aliquoted, and stored at −70 °C. Protein samples (40 µg/lane) were loaded onto a 10% SDS–PAGE gel and then electroblotted onto a nitrocellulose membrane for 1 h at 350 mA at 4 °C. Incubation with a blocking solution containing 5% skimmed milk powder in Tris-buffered saline with 0.2% Tween20 was performed for 2 h at room temperature. Then, the blots were incubated overnight at 4 °C with a primary antibody: rabbit anti-Nrp1 (1:1000, Abcam, Cambridge, UK) or mouse anti-GAPDH protein (1:10,000, Chemicon, Darmstadt, Germany). After washing, the blots were incubated for 2 h at room temperature with the goat anti-rabbit (1:7000, BioRad Laboratories Hercules, CA, USA) or goat anti-mouse (1:10,000, Chemicon, Darmstadt, Germany) secondary antibodies conjugated with horseradish peroxidase. Detection was performed using WesternBright ECL (Advansta, San Jose, CA, USA). Images were taken and analyzed using Gene Tools 2.1. software on a G:BOX.A (Syngene, Cambridge, UK).

### 4.8. Data Analysis and Statistics

Images from the Nissl-stained and myelin-stained tissues were obtained using a Nikon Eclipse 90i microscope connected to a computer with Neurolucida 9.145 software (MBF Bioscience, Williston, VT, USA). Images of the immunofluorescent brain sections, including double immunolabaled BrdU and CC1, or Olig2 and DAPI, were captured with a confocal spinning-disk microscope (Zeiss, Oberkochen, Germany). Double-labeled cells were counted, and for statistical analysis, a one-way analysis of variance (ANOVA) was conducted; this was followed by a Brown–Forsythe test. The Nrp1 protein levels were analyzed with one-way ANOVA in GraphPad Prism 8.4.3. Differences were considered significant at *p* < 0.05.

## 5. Conclusions

Here, we have demonstrated that the upper-layer neurons of the opossum, *Monodelphis domestica*, express Satb2 and neuropilin 1 interacting protein, which are proteins essential for the formation of the corpus callosum in eutherians. This indicates that extrinsic, but not intrinsic, cues should be a key player in guiding the axons of newly generated cortical neurons. We also detected that a population of glial cells is present in the midline of the anterior commissure. These glial cells act as a substrate for fibers crossing the midline, like a glial wedge in eutherians, and this is most likely the reason why marsupials do not form a glial wedge. Extracellular factors that exert their effects on axons are important for the proper development of cortical pyramidal neuron axons and their pathfinding. Such research will help create a corpus callosum in the opossum. However, the main technical limitation is the lack of a transgenic opossum. The use of genomic and proteomic approaches to identify these molecules is the next step towards understanding the transcription program in developing pyramidal neurons whose axons project to the anterior commissure. Future research in this direction may help develop methods for assessing the risk of occurrence and the diagnosis of developmental disorders of the corpus callosum in humans.

## Data Availability

The datasets used and analyzed during the current study are available from the corresponding author upon reasonable request.
